# The Hon. Sydney Holland's Latest Circular

**Published:** 1905-06-17

**Authors:** 


### THE HON. SYDNEY HOLLAND'S LATEST CIRCULAR.

### "THE FACTS SPEAK FOR THEMSELVES."

THE Hon. Sydney Holland has issued from the London Hospital to the Chairman and Secretaries of Hospitals and other persons whom he considers to be interested in the matter, a circular leaflet attacking the advertising system of THE HOSPITAL. Our system is already well known and understood by those whom Mr. Holland, with less accurate knowledge, purports to enlighten.

Mr. Holland misleads by contrasting matters which do not afford material for contrast or comparison. He contrasts the charges made for advertisements in our Appeal Supplements, which are wholly reserved for Hospitals and Institutions, with a scale which is the ordinary scale for advertisements in THE HOSPITAL but which he erroneously describes as the scale for advertisements in the Autumn and Spring numbers of THE HOSPITAL, the numbers being open to Traders as well as to Hospitals and Institutions.

The charges made for advertisements in our Appeal Supplements do not meet the cost of production of those Supplements, as is shown by the Auditor's certificate printed at the foot of this Note. The Certificate disposes of Mr. Holland's reckless statement that "the extortionate charges made for advertising in it (the Appeal Supplement) is to bring 'grist to the Mill' of the Proprietor or Proprietors of THE HOSPITAL."

The Appeal Supplement is designed to give to Hospitals and Institutions an opportunity of sending out their Appeals in a form in which they are not obscured by a cloud of trade advertisements, but are grouped so as to meet most readily the eye of readers who are seeking for such information. For this reason Trade Advertisements, which, if admitted, would quickly change a loss into a profit, are rigorously excluded. The loss on the production and issue of the Appeal Supplement is one of our contributions to Hospitals in general.

For advertisements in the ordinary Numbers and in the Spring and Autumn Numbers of THE HOSPITAL, which are open to Hospitals and Institutions and to Traders, the charges to Hospitals and Institutions are by a considerable percentage lower than those made to Traders. In our advertisement business, as in that of every other newspaper, special arrangements of course have to be made from time to time with individual advertisers, the magnitude and continuity of whose advertisements entitles thom to require special rates. This applies to Hospitals and Institutions as well as to Traders.

Mr. Holland should know, as the fact is, that the Hospital from which he writes, and which advertises in THE HOSPITAL, pays through an agent a lower rate than that which he quotes as charged to Tradesmen, the London Hospital having stipulated that their advertisement should always occupy a position of special prominence.

CERTIFICATE OF AUDITOR AND
MESSRS. DELOITTE, DEVER, GRIFFITHS AND CO.

To the Proprietors of THE HOSPITAL,
28 & 29 Southampton Street, W.C.

I have examined the accounts relating to the publication of the Hospital Sunday and Christmas Appeal Supplements of THE HOSPITAL newspaper for the five years from 1900 to 1904 inclusive, and find the result to be a loss to the proprietors.

No advertisements from traders are accepted for insertion in these supplements.

T. TURKETINE,
(Craggs, Turketine and Co.),
Auditor of The Scientific Press, Limited.
52 Coleman Street, London, E.C.
June 14th, 1905.

We confirm the above.

DELOITTE, DEVER, GRIFFITHS AND CO.,
Chartered Accountants,
4 Lothbury, E.C.

### THE CIRCULAR AND THE CANVASSER'S LETTER.

THE following is the circular referred to which the Honourable Sydney Holland writing from the London Hospital, E., under date June 1905, has widely distributed, though he has not favoured THE HOSPITAL with a copy of it:—

DEAR SIR,

You will probably be receiving, if you have not already received, a letter from the advertising canvasser of THE HOSPITAL newspaper, pointing out to you the advantages of advertising in THE HOSPITAL Sunday Supplement of that paper.

I have received such a letter, written to the Poplar Hospital, in which we are told that "*the sole design of this Supplement is to serve the Voluntary Institutions, and consequently the charges which are made for appeals are nominal.*"

I take leave to say that this is nothing short of inaccurate and misleading humbug, and I hope the letter has not been seen by the owner or owners of THE HOSPITAL newspaper.

The object of this Supplement, and, as I think, the extortionate charges made for advertising in it, is to bring "grist to the mill" of the proprietor or proprietors of that paper. There is no reason why this should not be done, but in face of what I am about to tell you, it is too much to suggest that the charges made are on such a scale as to make the insertion of these advertisements an act of disinterested philanthropy!

And to say that the charges are "nominal" is to state what is untrue.

I wonder whether Hospital Chairmen and Secretaries know that this paper, whose "sole design is to serve the Voluntary Hospitals," charges Hospitals very greatly in excess of what is charged to Tradesmen for the same amount of space. I wonder, also, if they know that Hospitals are not even allowed the special reduction which is made to Tradesmen, even on the lower prices charged to them, if they send their advertisements through an agent.

I think that when Hospital officials know this they will, if they continue to advertise at all, rightly insist that they should be put, as regards advertisements, on the same scale of charges as Tradesmen who advertise in that paper. Consider the huge disproportion of charges made "solely in the interests of the Voluntary Institutions." There are four special issues of The Hospital in the course of the year, called—

The Spring Number.
The Autumn Number.
The Hospital Sunday Number.
The Christmas Number.

Each of these issues is said to be about 25,000 to 30,000, so that, if the paper really ever reaches that number of readers, the numbers are about the same.

Here are the prices charged. They have varied slightly in the course of the last few years, but generally in the direction of raising them, rather than lowering them, to hospitals.

| | *Charge to Hospitals. *Hospital Sunday. Christmas Appeal.* | Charge to Tradesmen. *Autumn Number. Spring Number.* | If through an Agent. |
|---|---|---|---|
| 1 page ... ... | £7 7 0 | £5 0 0 ... | £4 0 0 |
| ½ page ... ... | 3 15 0 | 2 15 0 ... | 2 0 0 |
| ¼ page ... ... | 2 0 0 | 1 10 0 ... | 1 0 0 |
| ⅙ page ... ... | 1 12 0 | — | — |
| ⅛ page ... ... | 1 5 0 | 0 15 0 ... | 0 10 0 |

* These are the lowest charges to Hospitals. I have a letter from the agent quoting even a higher scale.

Hospitals are therefore charged very much more for the same amount of space than is charged to tradesmen.

*The Lancet*, whose sole design is really to help Hospitals, delivers a large number of free copies in the Churches, and makes no charge at all to Hospitals, though setting out the figures of each separately.

*We are told in this same letter, issued from the office of* The Hospital, *that* The Hospital *has the privilege of being the only paper delivered in St. Paul's Cathedral.* And this is held out to us as a further inducement to pay these "nominal" charges for advertising in it.

I shall be surprised if the authorities of St. Paul's Cathedral continue this privilege, when they learn that it is used as a lever to get advertisements at this high rate from hospitals whom they (the authorities) certainly wish to help.

I have had a correspondence with Sir Henry Burdett about this disproportion of charges made to hospitals and tradesmen, and he has denied absolutely that hospitals are charged more for the same amount of space. But the facts speak for themselves. Yours faithfully,

Sydney Holland.

### THE CANVASSER'S CIRCULAR.

The following is the circular letter of the advertising canvasser, Mr. G. Owen Ryan, to which Mr. Holland refers. It will be seen that Mr. Holland has misquoted and has given a meaning to Mr. Ryan's statements which they do not in fact bear. See the statements in italics:—

Dear Sir,—

On the occasion of Hospital Sunday, a special supplement will be issued to assist the charities, with a circulation of about 30,000 copies. *It will be distributed at St. Paul's Cathedral (by exclusive permission) and at the principal churches and chapels in London,* on the Sunday preceding Hospital Sunday, and therefore being in advance of the collection, and moreover at the height of the London season, it will constitute the most prominent and influential means that could be employed of inducing direct support.

This journal is, as you know, widely known as representative of the institutions. It has the confidence of the public, and also of the Press, who regularly quote it, and advise the public to be guided by it. It consequently is the only special journal that can lend a useful influence to appeals, and therefore it is best calculated to serve your ends.

I trust you will sanction the insertion of your announcement as before, and may remark that the sole design of this Supplement is to serve the voluntary institutions, and consequently the charges which are made for appeals are nominal,* and do not meet the outlay that is involved. I await the favour of your instructions, and shall be glad if at the same time you will send me particulars in brief of your current needs for editorial purposes.

* *Note.*—Mr. Ryan is justified in applying the word "nominal" (see definition in the Century Dictionary) to the scale of charges for advertisements in these Supplements, to hospitals and charities as the certificate of Mr. Turketine of Messrs. Craggs, Turketine and Co., the auditor, supported by that of the eminent firm of accountants, Messrs. Deloitte, Dever, Griffiths and Co., after an examination of the books proves that the cost of production of the Appeal Supplements is not met by the amounts charged for advertisements.

